# miR-29b as an Anti-Fibrotic Therapeutic: Mechanisms, Disease Biology and Translational Opportunities

**DOI:** 10.3390/cells15161472

**Published:** 2026-08-17

**Authors:** Lee Armstrong, Declan J. McKenna, Eva Mihalovova, Roise D. Gribben, Anton W. Roodnat, Bridgeen Callan, Colin E. Willoughby

**Affiliations:** 1 Centre for Genomic Medicine, Ulster University, Coleraine BT52 1SA, UK; 2 School of Pharmacy and Pharmaceutical Sciences, Ulster University, Coleraine BT52 1SA, UK

**Keywords:** miR-29b, fibrosis, microRNA, fibro-miR, extracellular matrix, TGF-β, collagen maturation, target validation, tissue-targeted delivery, ocular fibrosis

## Abstract

**Highlights:**

**What are the main findings?**

- miR-29b is a conserved anti-fibrotic regulator that suppresses extracellular matrix synthesis, collagen maturation and pro-fibrotic signalling across multiple organs.
- Therapeutic efficacy of miR-29b mimics is influenced by cell type, disease stage, target validation and tissue-selective delivery, with cardiac effects remaining context dependent.

**What are the implications of the main findings?**

- miR-29b replacement is most compelling for matrix-producing cells and locally accessible tissues, but direct target evidence and mechanistic biomarkers should guide development.
- Clinical translation will require scalable delivery, pharmacokinetic control, repeat-dose safety and preservation of normal wound healing and tissue homeostasis.

**Abstract:**

Fibrosis emerges when normally self-limiting tissue repair fails to resolve and overlapping phases of injury, stromal activation, extracellular matrix (ECM) deposition and remodelling become sustained. MicroRNAs (miRNAs) shape this transition by coordinating signalling, cell-state and matrix programmes. Functionally, pro-fibrotic fibro-miRs amplify fibrogenic pathways, whereas anti-fibrotic miRNAs restrain fibroblast activation and ECM production; the miR-29 family is a principal member of the latter group. This review examines miR-29 family organisation, the regulation of miR-29b by transforming growth factor-β (TGF-β)/Smad and additional transcriptional and inflammatory inputs, and the molecular targets through which miR-29b controls collagen synthesis, processing and crosslinking. Direct canonical targets are distinguished from experimentally supported, predicted and indirect pathway components. Evidence is evaluated across fibroblasts and myofibroblasts, epithelial and endothelial cells, and pulmonary, hepatic, renal, cardiac, dermal and ocular fibrosis models. Therapeutic translation is considered in relation to miR-29b mimics and agomirs, local and tissue-targeted delivery, pharmacokinetics, dose control, off-target repression, immune activation and long-term safety. Overall, miR-29b remains a credible network-level anti-fibrotic candidate, but successful translation requires cell- and disease-specific target validation, selective delivery and preservation of physiological wound repair.

## 1. Introduction

Fibrosis is a pathological process shared across many chronic diseases [1]. It is characterised by excessive extracellular matrix (ECM) deposition, persistent fibroblast activation, tissue stiffening and progressive disruption of normal tissue architecture [2]. Fibrosis affects several organs, including the lung, liver, kidney, heart and skin, and similar processes in ocular tissues [3]. Importantly, fibrosis is not simply the accumulation of collagen [4]. It involves increased matrix production, altered matrix degradation, collagen crosslinking, inflammation, myofibroblast activation and changes in tissue mechanics [5]. These processes gradually reduce normal tissue function and can contribute to organ failure [6]. Although anti-fibrotic treatments are available for some diseases, current approaches are often organ specific and usually slow disease progression rather than reverse established fibrosis [7].

From a therapeutic perspective, the key issue is that fibrotic remodelling is sustained by overlapping pathways that regulate matrix production, cell contraction, inflammation and tissue stiffness [8,9]. Transforming growth factor-β signalling is one of the central drivers of this process, but it acts within a wider network that includes ECM assembly, collagen maturation, integrin signalling, growth factor activity and mechanical feedback from the stiffened matrix [10,11]. As fibrosis progresses, these pathways reinforce each other [12,13]. Increased matrix deposition stiffens the tissue, altered mechanics further activate fibroblasts, and activated fibroblasts continue to produce and remodel the ECM [14,15]. This creates a self-sustaining pro-fibrotic state that is difficult to interrupt with therapies directed at only one downstream gene or pathway [16].

MicroRNAs (miRNAs) are well suited to this network-level problem because each mature miRNA can regulate multiple functionally related messenger RNAs (mRNAs) through the RNA-induced silencing complex (RISC) [17,18,19,20,21]. In fibrosis, miRNA dysregulation can alter fibroblast activation, inflammatory signalling, epithelial and endothelial plasticity, and the balance between ECM synthesis, processing and degradation. The same miRNA may have different consequences according to cell type, disease stage and target availability; functional labels such as pro-fibrotic or anti-fibrotic therefore describe the prevailing phenotype in a defined context rather than an immutable molecular taxonomy [18,22,23].

Previous work from this group has also shown that miRNA-mediated regulation of epithelial-to-mesenchymal transition (EMT) is relevant to disease biology, including miR-143-3p regulation of AKT serine/threonine kinase 1 (AKT1) and miR-145-5p regulation of myosin VI (MYO6) in prostate cancer, alongside thesis work linking miRNA dysregulation with EMT in prostate cancer and glaucoma [24,25,26]. A miRNA that represses multiple ECM and pro-fibrotic targets could therefore have broader regulatory effects than a conventional single-target approach [21].

The miR-29 family has emerged as one of the most consistently fibrosis-associated miRNA families [27,28,29,30]. Within this family, miR-29b has received particular attention because it is repeatedly downregulated in fibrotic tissues and because restoring its expression reduces pro-fibrotic gene expression in experimental models [28,31]. In healthy tissues, miR-29b contributes to matrix homeostasis by limiting expression of ECM and matrix-associated genes [17,19]. During fibrosis, pro-fibrotic signals, particularly TGF-β/Smad signalling, suppress miR-29 expression [29,32]. This reduction in miR-29b expression removes an important post-transcriptional brake on matrix production [27,28]. As a result, expression of genes involved in collagen synthesis, matrix organisation, crosslinking and fibroblast activation can increase more readily [33,34].

This relationship places miR-29b at an important point in fibrotic regulation. It is not simply a marker of fibrosis, nor is it best understood as a regulator of one isolated target. Instead, miR-29b acts as a network-level suppressor of fibrotic remodelling [28,32]. Experimental studies across several organs show that increasing miR-29b can reduce collagen expression, limit myofibroblast features and decrease ECM accumulation [29]. These findings have also led to therapeutic interest in miR-29b replacement strategies, including chemically modified miRNA mimics and tissue-targeted delivery systems [27,28]. Early clinical work in skin fibrosis has provided proof-of-concept that miR-29 mimic therapy can reduce ECM gene expression in humans, although broader clinical translation remains at an early stage [35].

This review first establishes the biological framework that separates successful wound repair from pathological fibrosis and positions miRNAs within that network. It then outlines miR-29 family organisation and biogenesis, examines upstream control of miR-29b, and evaluates direct targets separately from supported or indirect regulatory associations. Cellular mechanisms are considered across fibrogenic, epithelial and endothelial compartments, followed by organ-specific evidence and key issues relating to therapeutic replacement, delivery, clinical translation, safety, controversies and future priorities.

## 2. Biological Framework of Fibrosis and the miRNA Network

### 2.1. Overlapping Phases of the Fibrotic Response

Fibrosis can be organised into four overlapping phases: initiation, stromal activation, ECM deposition and remodelling or resolution [1,2,3,6,14]. This sequence is an analytical framework rather than a rigid chronology; inflammatory, cellular and matrix events frequently coexist, particularly in chronic disease.

During initiation, tissue injury activates coagulation, damage-associated molecular pattern signalling and the release of cytokines and growth factors from injured parenchymal cells, platelets, immune cells and the provisional matrix [1,6]. These signals recruit inflammatory cells and establish a local environment rich in TGF-β, platelet-derived growth factor and other mediators that initiate stromal responses.

During activation, resident fibroblasts and organ-specific stromal populations, including hepatic stellate cells and perivascular cells, proliferate, migrate and acquire contractile myofibroblast features [2,9,14]. Activated cells express alpha-smooth muscle actin (α-SMA), reorganise actin stress fibres, increase contractility and become major sources of ECM. The relative contribution of each precursor population is organ- and model-dependent and should not be inferred from marker expression alone [2,36].

During deposition, activated stromal cells produce fibrillar and basement membrane collagens, fibronectin, proteoglycans and matricellular proteins [4,5]. Scar formation also depends on collagen folding, secretion, propeptide processing, fibril assembly and enzymatic crosslinking; these maturation steps determine the stability and mechanical properties of the deposited matrix [4,14,37].

During remodelling, matrix metalloproteinases, their inhibitors and collagen-crosslinking enzymes determine whether the provisional scar is degraded, reorganised or stabilised [5,6]. Successful resolution includes inflammatory withdrawal and loss or deactivation of myofibroblasts. Persistent signalling, apoptosis resistance and mechanical reinforcement by a stiff matrix instead convert a reparative response into progressive fibrosis [9,14,38,39].

### 2.2. Physiological Wound Healing Versus Pathological Fibrosis

Physiological repair and pathological fibrosis use many of the same pathways, but they differ in duration, feedback control and outcome. Homeostatic healing terminates once tissue integrity is restored; fibrosis represents failed resolution in which stromal activation and matrix remodelling remain self-sustaining [1,2,3,6]. The main distinctions are summarised in Table 1.

### 2.3. General Roles of miRNAs in Fibrosis

After processing by Drosha and Dicer, mature miRNAs guide Argonaute-containing RISC to partially complementary sites in target transcripts, usually within the 3′ untranslated region (3′UTR), leading predominantly to mRNA destabilisation and reduced protein output [19,20,21,22,40,41,42]. Because target recognition is sequence- and context-dependent, a miRNA can regulate several nodes within one biological programme without affecting every predicted transcript in every cell.

Within the fibrotic network, miRNAs influence fibroblast-to-myofibroblast differentiation, cell migration, proliferation, survival and contractility; TGF-β, Wnt, PI3K/AKT, MAPK and inflammatory signalling; ECM synthesis, processing and degradation; epithelial-to-mesenchymal transition (EMT) and endothelial-to-mesenchymal transition (EndMT)-associated programmes; and paracrine communication among stromal, epithelial, endothelial and immune cells [18,43,44,45]. These effects explain why miRNA dysregulation can alter both the quantity of the matrix and the cellular state that sustains its production.

### 2.4. Functional Classification of Fibrosis-Associated miRNAs

A practical classification divides fibrosis-associated miRNAs according to their dominant function in a defined model. Pro-fibrotic fibro-miRs amplify fibrogenic signalling or repress endogenous inhibitors, whereas anti-fibrotic miRNAs restrain ECM production, activation or phenotypic transition. A third context-dependent group is needed because the same miRNA may act differently across cell types or disease stages. This system is functional, not a formal taxonomic hierarchy. Representative examples are summarised in Table 2.

**Figure 1:**
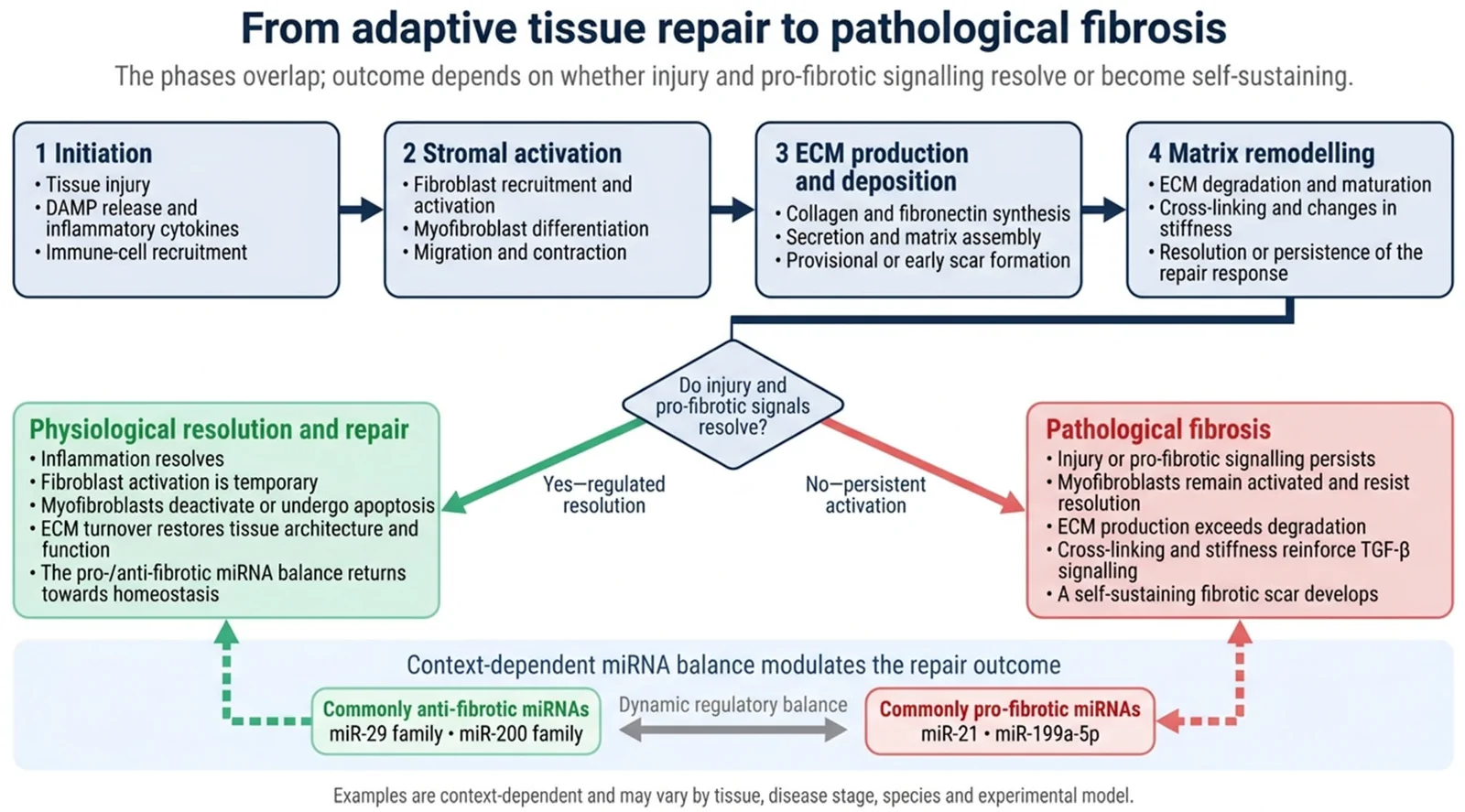
Conceptual framework linking the overlapping phases of tissue repair to either resolution or pathological fibrosis. Temporary inflammation, stromal activation and ECM deposition permit healing, whereas persistent myofibroblasts, excessive crosslinking and matrix stiffness create a self-reinforcing scar. The functional balance between pro-fibrotic fibro-miRs and anti-fibrotic miRNAs contributes to this divergence [1,2,3,4,5,6,14,27,28,33,43,44,45]. Solid arrows indicate progression through repair and branching to resolution or fibrosis; dashed arrows indicate miRNA-mediated regulatory influence on these outcomes; the grey bidirectional arrow denotes the dynamic balance between anti-fibrotic and pro-fibrotic miRNAs.

Within this framework, the miR-29 family is one of the best-supported anti-fibrotic miRNA families because it directly represses multiple ECM transcripts and regulates components of matrix maturation. Its recurrent downregulation across fibrotic organs distinguishes it from miRNAs whose effects are confined to a single tissue, while the cardiac literature illustrates why context and family-member specificity must still be retained [27,28,29,30,31,32,33,48]. This framework is illustrated in Figure 1.

## 3. The miR-29 Family: Genomic Organisation and Biogenesis

The miR-29 family consists of three mature miRNAs: miR-29a, miR-29b and miR-29c [47,50]. These molecules are closely related but are not identical [30,47]. They share sequence similarity, particularly within the seed region that guides target recognition, and this means they often regulate overlapping sets of mRNAs [51,52]. For this reason, many studies discuss miR-29 activity at the family level [29,30].

However, miR-29b is also commonly examined as a distinct molecule because it has specific expression patterns, regulatory features and experimental evidence in fibrosis models [47,53]. This distinction is important for interpreting the literature, because some studies measure or manipulate the whole miR-29 family, whereas others focus specifically on miR-29b [27,30,46].

The miR-29 family is encoded by two genomic clusters [47,50]. One cluster contains miR-29b-1 and miR-29a, while the second contains miR-29b-2 and miR-29c [47,54]. This arrangement means that miR-29b is represented in both genomic loci, whereas miR-29a and miR-29c are each associated with one of the two clusters [30,54]. The clustered organisation also supports coordinated regulation of family members, although the mature miRNAs may still differ in abundance, stability and cellular localisation [30,47,55]. In practical terms, this genomic organisation helps explain why pro-fibrotic or anti-fibrotic signals can alter more than one miR-29 family member at the same time [30,47,56].

Like most canonical miRNAs, miR-29 family members are generated through the Drosha/Dicer processing pathway [20,57]. The miR-29 genes are first transcribed as primary miRNA transcripts [20,58].

These primary transcripts are processed in the nucleus by the RNase III enzyme Drosha to produce precursor miRNAs [20,57]. The precursor molecules are then exported to the cytoplasm, where Dicer cleaves them into mature miRNA duplexes of approximately 22 nucleotides [20,22]. One strand is then incorporated into the RNA-induced silencing complex (RISC) [22,58]. Once loaded into RISC, mature miR-29b acts as a guide molecule that directs the complex to target mRNAs [22,58].

For miR-29b specifically, strand identity should be stated because the two precursors can generate hsa-miR-29b-1-5p, hsa-miR-29b-2-5p and the shared hsa-miR-29b-3p mature strand. The 3p product is generally the more abundant mature form and is the strand most commonly meant when studies refer to miR-29b in fibrotic regulation. The 5p products may differ in seed sequence and precursor origin, so they should not automatically be assumed to have the same target mRNA repertoire or biological effects as miR-29b-3p [30,32,47].

Target recognition is mainly determined by sequence pairing between miR-29b and complementary regions in target mRNAs [19,51]. In most cases, this interaction occurs within the 3′ untranslated region (3′UTR) of the target transcript [58,59]. Binding does not usually require perfect complementarity [19,40]. Instead, partial pairing, especially through the seed region of the miRNA, is often sufficient to reduce gene expression [51,52]. This repression can occur through reduced mRNA stability, reduced translation, or both [40,41,42].

Some studies also describe non-canonical miR-29 binding sites, including sites within coding regions, but 3′UTR-mediated repression remains the main mechanism used to explain most validated miR-29 targets [23,54,60].

This mode of action is central to how miR-29b influences cellular phenotype [17,18]. A single mature miRNA can repress several transcripts that belong to related biological pathways [17,22]. However, this also means that the target assignment must be interpreted carefully [23,61]. A decrease in a candidate target after miR-29b overexpression does not by itself prove direct binding [61,62]. Stronger evidence comes from approaches such as 3′UTR reporter assays, mutational analysis of the binding site, and rescue experiments [61,62,63]. This distinction becomes important later in the review, where validated targets need to be separated from predicted or indirectly regulated genes [52,61,63].

The relationship between miR-29a, miR-29b and miR-29c also affects how the evidence base should be evaluated [30,47]. Because the family members share a related seed sequence, they can regulate many of the same transcripts [47,51,52]. Therefore, findings reported for “miR-29” may be relevant to miR-29b but should not automatically be treated as miR-29b-specific evidence [30,47,53]. Conversely, studies that directly manipulate miR-29b provide clearer evidence for the role of this individual family member [54,61]. This review therefore distinguishes, where possible, between family-level observations and evidence specifically involving miR-29b [30,47].

There are also features that support separate consideration of miR-29b [32,55,64]. Human cells express two forms of miR-29b that differ by one nucleotide at the 3′ end, while retaining the same core seed sequence [47,64]. In addition, miR-29b contains a hexanucleotide terminal motif associated with nuclear localisation; mutation or deletion of this motif can impair nuclear import and increase cytoplasmic localisation, potentially strengthening canonical cytoplasmic target repression [32,55]. Sequence-level differences may also influence stability: miR-29b and miR-29c contain a tri-uracil sequence at positions 9–11 that has been linked to more rapid decay compared with miR-29a, which contains a cytosine at position 10 [32]. These features do not replace the canonical model of miRNA function, but they indicate that miR-29b biology may be more complex than simple family-level target overlap [22,23,64].

Under normal conditions, miR-29 family members contribute to the control of ECM gene expression and help maintain matrix balance [27,34,65]. In fibrotic disease, this balance is disrupted when pro-fibrotic pathways suppress miR-29 expression [28,46,66]. The next section therefore examines the regulation of miR-29b in fibrotic signalling, with particular emphasis on TGF-β/Smad-mediated repression [10,46,66].

## 4. Upstream Regulation of miR-29b in Fibrotic Signalling

miR-29b is not only altered during disease, it is also part of normal tissue regulation [30,32]. Under homeostatic conditions, members of the miR-29 family are expressed across a range of tissues and contribute to ECM control [30,47]. Their basal expression helps restrain inappropriate matrix gene expression and supports normal matrix turnover [5,34]. This is important because most adult tissues require continuous ECM maintenance, but excessive matrix synthesis or reduced matrix degradation can shift the tissue toward fibrosis [4,16]. In this context, miR-29b functions as part of the normal post-transcriptional system that limits matrix accumulation [19,21].

### 4.1. TGF-β/Smad-Dependent Repression and Mechanical Feedback

During fibrosis, the regulatory balance of the ECM is disrupted [2,3]. One of the most consistent mechanisms is suppression of miR-29 expression by TGF-β signalling [46,66,67]. TGF-β is a central pro-fibrotic cytokine in many tissues and promotes fibroblast activation, myofibroblast differentiation and ECM production [10,12]. In several fibrotic models, TGF-β signalling through SMAD family member 3 (Smad3) reduces expression of miR-29 family members, including miR-29b [11,46]. This has been reported in contexts such as renal fibrosis, pulmonary fibrosis, hepatic stellate cell activation and cardiac remodelling [27,28,66,68]. The result is a reduction in miR-29-mediated repression at the same time that pro-fibrotic transcriptional programmes are being activated [22,46]. In glaucoma-relevant cell assays, pharmacological Smad3 inhibition could also serve as a useful pathway control to determine whether miR-29b replacement reproduces, complements or differs from direct blockade of TGF-β/Smad signalling.

The biological consequence of this miR-29-mediated repression is important [29]. When miR-29b levels fall, ECM and matrix-associated transcripts are less strongly restrained [28,33]. This allows increased expression of genes involved in collagen production, matrix assembly, matrix stabilisation and fibroblast activation [4,27]. The key point is not that miR-29b controls one fibrosis gene, but that its loss can release several components of the fibrotic programme concurrently [2,29]. This explains why reduced miR-29b expression is frequently associated with increased matrix deposition in fibrotic tissues [33,68].

TGF-β-mediated repression of miR-29b can also contribute to a feed-forward fibrotic loop [6,38]. Increased TGF-β activity suppresses miR-29b [66,69]. Reduced miR-29b then permits higher matrix gene expression [28,47]. As ECM accumulates, tissue stiffness and altered cell–matrix signalling can further activate fibroblasts and maintain pro-fibrotic signalling [9,39]. In this model, miR-29b loss is not simply a downstream marker of fibrosis [36,68]. It contributes to the persistence of the fibrotic state by weakening the endogenous brake on matrix production [46,69].

### 4.2. Additional Transcriptional, Inflammatory and Anti-Fibrotic Inputs

TGF-β/Smad3 is the most reproducible upstream repressor in fibrosis, but the MIR29 network integrates additional inputs. c-Myc, Hedgehog signalling and nuclear factor-κB (NF-κB) can repress the miR-29b-1/miR-29a promoter, providing a direct link between proliferative or inflammatory signalling and reduced miR-29 output [70]. Conversely, hepatocyte growth factor induces miR-29 in hepatic stellate cells and reduces collagen I and IV synthesis, while BMP-7 and Smad7 provide inhibitory counter-regulation of renal TGF-β/Smad signalling [69,71,72,73].

Other pathways should be framed according to the direction and strength of evidence. PI3K/AKT and Wnt/β-catenin interact reciprocally with miR-29 biology in selected liver and cardiac models [48,74], but these data do not establish either pathway as a universal direct repressor of MIR29B transcription. Similarly, oxidative stress, inflammatory persistence and matrix stiffness can sustain TGF-β, NF-κB and mechanotransduction signals that correlate with miR-29 loss, whereas direct promoter occupancy or causal epigenetic regulation are not demonstrated in every tissue [38,39,70,75]. Long non-coding and circular RNA ‘sponges’ have been proposed in several disease settings, but they should be treated as emerging, context-specific modulators unless stoichiometric sequestration and functional rescue are shown.

The resulting model is therefore a regulatory network rather than a single linear pathway: established direct transcriptional inputs converge on miR-29 expression, while downstream ECM and signalling changes feed back through tissue mechanics and cytokine activation. Distinguishing direct promoter regulation from pathway crosstalk prevents associations from being presented as equivalent mechanisms.

### 4.3. Cell Type, Disease Stage and Reciprocal Regulation

Regulation of miR-29b is also likely to be tissue- and cell-type-dependent [8,36]. Fibroblasts, epithelial cells, hepatic stellate cells, endothelial cells, smooth-muscle-like cells and resident stromal cells may not regulate miR-29b in identical ways [1,3]. Disease stage may also matter [2,6]. Early fibrosis, active inflammatory fibrosis and established scar tissue may each involve different levels of miR-29b repression and different downstream consequences [2,6]. This is one reason why some studies report family-level changes in miR-29a, miR-29b and miR-29c together, whereas others identify miR-29b-specific effects [30,47].

This context dependence is important for interpreting the literature [29,30]. In most fibrotic systems, reduced miR-29b aligns with increased matrix production and restoration of miR-29b has anti-fibrotic effects [28,31]. However, the strength of this relationship may differ between organs, cell types and experimental models [8,36]. Some findings, particularly in cardiac remodelling, suggest that miR-29 biology can be more complex than a single universal anti-fibrotic rule [27,48]. These exceptions do not negate the importance of miR-29b in fibrosis, but they show that therapeutic use will require tissue-specific validation [2,76].

**Figure 2:**
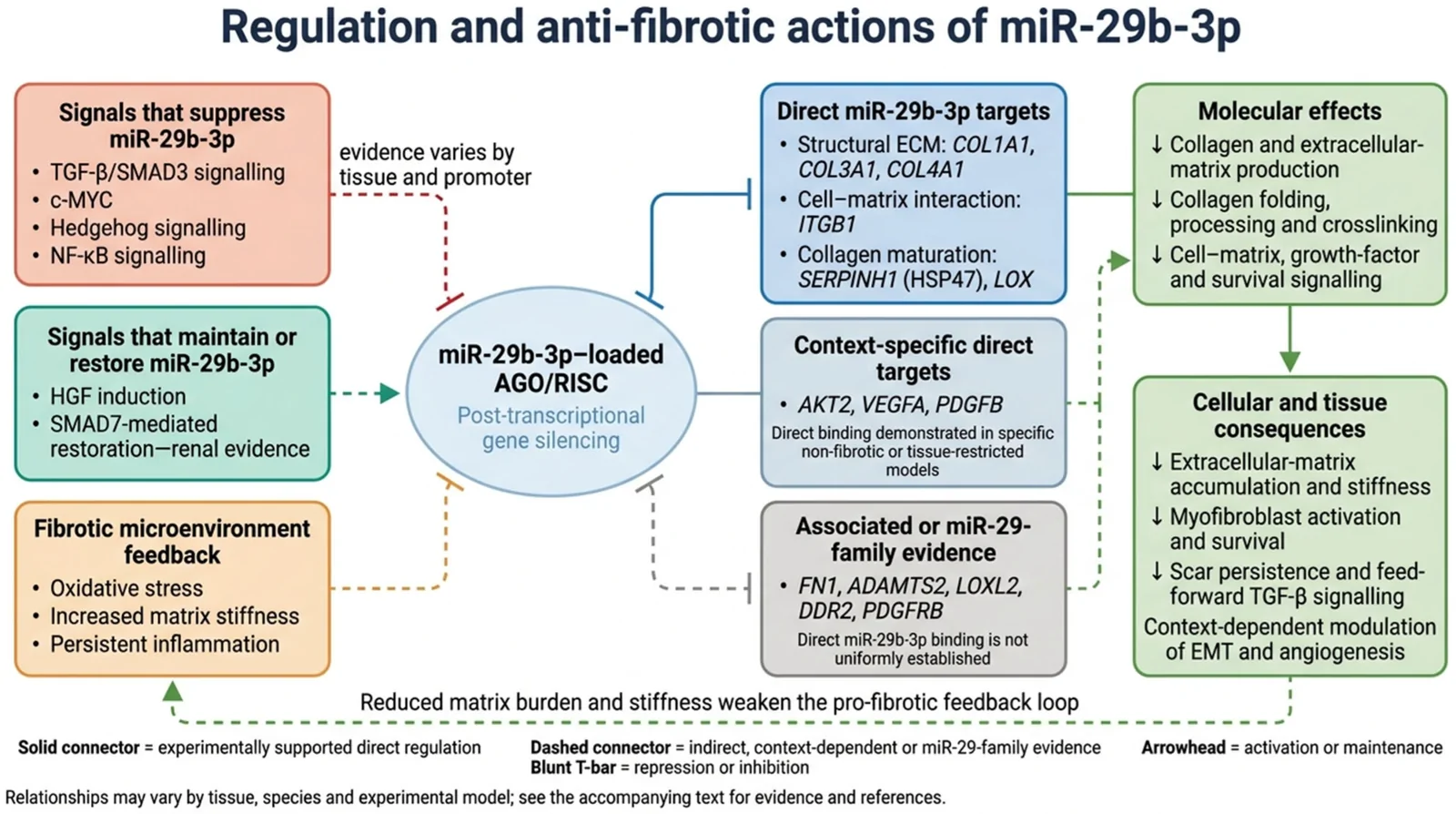
Mechanistic integration of upstream miR-29b regulation, molecular targets and downstream phenotypes. Solid connectors denote direct promoter or target-site evidence; dashed connectors denote indirect, reciprocal or context-dependent relationships. HSP47/SERPINH1 and LOX have miR-29b 3′UTR reporter and target-site deletion evidence in hepatic stellate cells, whereas ADAMTS2 and several adhesion-associated components remain supported by family-level associations. AKT2, VEGFA and PDGFB illustrate direct effects demonstrated in epithelial or endothelial models rather than universal fibrosis targets [37,46,66,69,70,73,74,77,78,79,80,81,82,83]. Blunt T-bars denote repression or inhibition, whereas arrowheads denote activation or maintenance.

Overall, miR-29b is regulated by a pro-fibrotic signalling environment in which TGF-β/Smad-mediated repression plays the leading role [46,66]. Loss of miR-29b weakens post-transcriptional control over matrix-associated genes and supports the persistence of fibrotic remodelling [4,22]. The next section examines the downstream molecular targets of miR-29b, distinguishing direct validated targets from genes that are predicted or indirectly regulated [61,62]. These relationships are summarised in Figure 2.

## 5. Molecular Targets and Cellular Anti-Fibrotic Mechanisms

### 5.1. Evidence Hierarchy for miRNA Target Assignment

The functional importance of miR-29b in fibrosis depends on the genes it represses [22,28]. Many fibrosis-associated transcripts decrease after miR-29b restoration, but these genes should not all be treated as direct targets [23,61]. A direct target requires evidence that miR-29b physically interacts with the transcript, usually through a predicted binding site in the 3′UTR [51,52]. Strong evidence has been provided by luciferase reporter assays, mutation of the binding site, and consistent reductions in both mRNA and protein after miR-29b overexpression [61,62]. Weaker evidence comes from expression correlation, bioinformatic prediction, or transcript reduction without binding-site validation [61,63]. This distinction is important because miR-29b can alter gene expression directly through RISC-mediated repression, but also indirectly by changing fibroblast activation, matrix signalling or upstream regulatory pathways [18,41,54]. A further caveat is that many luciferase reporter assays are performed in heterologous systems such as human embryonic kidney 293 (HEK293) or HeLa cells. These assays establish that a binding site can mediate miR-29-dependent repression, but they do not by themselves prove regulation in fibrotic target cells. For trabecular meshwork and other disease-relevant models, stronger evidence would combine mimic or inhibitor studies, mRNA and protein readouts, rescue experiments, and crosslinking and immunoprecipitation (CLIP)-based approaches such as enhanced CLIP (eCLIP) or Argonaute CLIP (AGO-CLIP) in the relevant cell type.

For consistency, this review uses four evidence labels. A direct canonical target has a functional miR-29-binding site supported by a reporter assay and preferably site mutation or deletion. An experimentally supported target changes after miR-29b manipulation at mRNA and/or protein level, but lacks complete target-site validation. A predicted target is based mainly on sequence or database evidence. An indirectly regulated pathway component changes downstream of miR-29b without evidence of direct transcript binding. Reporter assays in heterologous cells establish site functionality but should be complemented by rescue, Argonaute occupancy and protein-level experiments in the relevant fibrotic cell.

### 5.2. ECM Structural Proteins and Matrix Assembly

Collagens are the central structural targets linked to miR-29b [27,28,33]. Fibrotic tissues contain increased fibrillar and basement membrane collagens, and miR-29b has repeatedly been associated with reduced collagen expression in experimental models [4,28]. Among the best-supported targets are collagen type I alpha 1 chain (*COL1A1*) and collagen type IV alpha 1 chain (*COL4A1*), which encode collagen I and collagen IV components [66,69,77]. These genes are particularly relevant because collagen I is a major fibrillar scar protein, while collagen IV contributes to basement membrane remodelling [4,5]. Reporter-based evidence supports direct miR-29 regulation of these collagen transcripts, and miR-29b restoration also reduces collagen mRNA and protein expression in fibrotic cell systems [66,69,77]. Other collagen genes, including collagen type III alpha 1 chain (*COL3A1*) and additional fibrillar or basement membrane collagens, are also frequently reduced after miR-29 restoration, although the level of direct validation varies between studies [27,28,33]. miR-29b also regulates genes involved in matrix assembly, maturation and stabilisation [37,78]. This is important because fibrosis is not determined only by the amount of collagen produced [4,5]. The organisation, folding and crosslinking of matrix proteins strongly influence tissue stiffness and scar persistence [14,37]. Fibronectin 1 (*FN1*) contributes to matrix scaffold formation and can support pro-fibrotic signalling [5,78]. Lysyl oxidase-like 2 (LOXL2) promotes collagen crosslinking and matrix stiffening, making it a key matrix maturation target [79,80]. Serpin family H member 1 (*SERPINH1*), also known as heat shock protein 47 (HSP47), functions as a collagen-specific chaperone and supports proper collagen folding [37,80]. Together, these data support miR-29b as a regulator of both collagen abundance and the matrix-processing machinery that stabilises fibrotic tissue [37,78,80].

Fibronectin, elastin, *SPARC* and several cell-matrix receptors also change after miR-29 manipulation, but the evidence is heterogeneous [27,28,65,78]. These components should be interpreted as parts of a coordinated matrix programme rather than assumed to be equivalent direct targets. This distinction is especially important when a study measures family-wide miR-29 activity or reports only transcript-level changes.

### 5.3. Collagen Folding, Processing and Crosslinking

miR-29b regulates more than collagen abundance. HSP47, encoded by *SERPINH1*, is an endoplasmic-reticulum collagen chaperone required for correct triple-helix folding and secretion. ADAMTS2 is an extracellular procollagen N-proteinase that removes amino-terminal propeptides to permit mature fibril assembly. LOX oxidatively deaminates lysine residues in collagen and elastin, enabling covalent crosslinks that stabilise the matrix and increase tissue stiffness [4,5,37].

For HSP47 and LOX, miR-29b reduced mRNA and protein expression and inhibited reporters containing full-length 3′UTRs; deleting the candidate miR-29b sites abolished reporter repression. These data support direct canonical targeting in hepatic stellate cell models [37]. By contrast, ADAMTS2 was identified among miR-29-responsive matrix processing transcripts in pulmonary profiling, but definitive miR-29b target-site validation is lacking [28]. It should therefore be described as a reported regulated component, not a confirmed direct target. LOXL2 also promotes crosslinking, but the strongest direct reporter evidence is largely miR-29 family or miR-29a evidence from cancer-associated models rather than miR-29b-specific fibrotic cells [79,80].

This layered control is biologically important: simultaneous repression of collagen chains, chaperoning, propeptide processing and crosslinking can reduce both the amount of ECM produced and the efficiency with which it becomes an insoluble, mechanically active scar.

### 5.4. Fibroblasts, Myofibroblasts and Organ-Specific Stromal Cells

The phrase ‘restriction of fibrogenic cells’ should be resolved into defined phenotypes. In fibroblasts and hepatic stellate cells, miR-29b restoration reduces collagen secretion and activation-associated markers, including α-SMA and fibronectin; it also reduces receptor and adhesion programmes linked to proliferation, migration and matrix sensing [31,77,78,81]. In activated hepatic stellate cells, miR-29b can suppress *PIK3R1*/AKT3 signalling and promote apoptosis, indicating a cell-survival mechanism in addition to matrix repression [74]. These effects are model-specific and do not imply that miR-29b indiscriminately eliminates all fibroblasts.

The aggregate phenotype is therefore a reduction in myofibroblast differentiation or maintenance, decreased contractile and matrix-producing capacity, and reduced survival rates in selected activated populations. Normal quiescent fibroblast functions and reparative responses remain safety concerns, reinforcing the need for dose and tissue selectivity. Selected targets and evidence levels are summarised in Table 3.

### 5.5. Epithelial and Endothelial Compartments

Epithelial injury can sustain fibrosis through barrier failure, release of pro-fibrotic mediators and partial EMT-associated transcriptional programmes. In a silicosis model, miR-29b promoted mesenchymal-to-epithelial features and reduced EMT-associated fibrosis [84]. In retinal pigment epithelial cells, miR-29b directly targeted *AKT2* and attenuated TGF-β1-induced EMT markers [82]. These findings support epithelial-state regulation, but they do not establish that epithelial cells are a major quantitative source of fibroblasts in every organ.

Direct miR-29b-specific evidence in endothelial fibrosis is more limited. In retinal microvascular endothelial cells, miR-29b-3p directly repressed *VEGFA* and *PDGFB* and reduced proliferation and angiogenic behaviour [83]. This result supports effects on vascular and paracrine signalling, but it is not itself proof that miR-29b prevents EndMT or endothelial contribution to scar-forming cells. Endothelial barrier dysfunction, capillary rarefaction and EndMT remain relevant hypotheses that require lineage-aware, disease-specific validation.

## 6. Organ-Specific Evidence

The molecular target profile of miR-29b suggests that it should have anti-fibrotic effects, but target repression alone is not sufficient evidence for therapeutic relevance [7,61]. The stronger question is whether restoring miR-29b reduces fibrosis in cells, animal models and human tissue settings [2,6]. The evidence should therefore be interpreted at several levels: in vitro studies, animal disease models, human tissue correlations and clinical intervention studies [2,7]. Cell studies are useful for defining mechanisms, while animal models test whether these mechanisms alter tissue fibrosis [3,61]. Human tissue studies show disease relevance, but they are often correlative [2,7]. Clinical intervention studies provide the strongest translational evidence because they test whether miR-29 replacement can produce measurable anti-fibrotic effects in humans [7,35].

Across organ systems, a common pattern has been reported: miR-29b or the wider miR-29 family is reduced in fibrotic settings, while restoration of miR-29 activity decreases ECM gene expression and fibrotic markers [29,30]. However, not all evidence is equally strong [30,47]. Some studies specifically manipulate miR-29b, whereas others examine miR-29a, miR-29c or the family as a group [30,47]. In addition, some organs have mainly preclinical evidence, while others have early clinical data [35,76]. This section therefore evaluates the evidence by organ system, while avoiding the assumption that all tissues support the same level of confidence [2,47,48].

### 6.1. Pulmonary Fibrosis

Pulmonary fibrosis is one of the strongest disease areas supporting a role for miR-29b [28,31,76]. In fibrotic lung settings, miR-29 expression is reduced and ECM gene expression is increased [28,85,86].

This pattern has been reported in idiopathic pulmonary fibrosis and in experimental lung fibrosis models [28,31,85]. TGF-β suppresses miR-29 in lung fibroblasts, linking the regulatory mechanism discussed earlier to a disease-relevant cell type [28,87]. Loss of miR-29 activity in this setting is consistent with increased collagen expression, fibroblast activation and myofibroblast differentiation [28,31,86].

Restoration of miR-29 activity has anti-fibrotic effects in lung models [31,87,88]. In human lung fibroblasts, miR-29 replacement reduces collagen expression and limits pro-fibrotic features [31,76]. A miRNA agomir is a chemically modified oligonucleotide designed to mimic an endogenous mature miRNA with enhanced stability, uptake and in vivo persistence. Unlike a conventional transient culture mimic, an agomir is optimised for biological delivery, whereas an antagomir inhibits the corresponding miRNA. In bleomycin- and silica-induced fibrosis, miR-29b mimic or agomir approaches reduce collagen deposition, α-SMA expression and histological fibrosis [84,87,88].

The lung is also important from a translational perspective [76,89]. Pulmonary fibrosis is a major clinical target for anti-fibrotic therapy, and inhaled or lung-targeted delivery approaches are being developed for miR-29 replacement [76,89]. This does not yet establish clinical efficacy in idiopathic pulmonary fibrosis, but it places pulmonary disease among the most advanced areas for miR-29 therapeutic development [31,76]. The main limitation is that much of the current evidence remains preclinical, and long-term outcomes, delivery efficiency and patient selection remain unresolved [7,76].

### 6.2. Liver Fibrosis

Liver fibrosis provides strong mechanistic evidence for miR-29b, particularly through studies of hepatic stellate cells [68,78]. Hepatic stellate cells are central fibrogenic cells in the liver [90,91]. When activated, they acquire myofibroblast-like features and produce large amounts of ECM [90,91]. In this context, miR-29b is reduced, while collagen and activation-associated genes increase [68,77,78]. This makes hepatic stellate cells a clear model for studying how miR-29b loss contributes to fibrogenic cell activation [74,77,78].

Restoring miR-29b in hepatic stellate cells reduces collagen transcripts and activation markers [77,78]. This includes reduced *COL1A1*/*COL1A2*, α-SMA, *FN1*, *ITGB1* and PDGFRβ after miR-29b restoration, consistent with the target profile described earlier [74,77,78,81]. Importantly, this evidence is not limited to one collagen transcript [37,77,78]. It suggests that miR-29b can suppress a broader stellate-cell activation programme [74,78,81].

Animal models also support a role for miR-29b in liver fibrosis [68,74]. In carbon tetrachloride and bile duct ligation models, miR-29b expression falls as fibrosis develops, while forced miR-29b expression reduces fibrotic markers and collagen deposition [68,74]. The evidence in liver fibrosis is therefore strong at the preclinical level [68,74,78]. However, it remains less clinically advanced than in the skin and lung fields [35,76]. The main current value of the liver literature is mechanistic: it shows how miR-29b can restrain a defined fibrogenic cell type and reduce matrix accumulation in experimental fibrosis [74,77,78].

### 6.3. Renal Fibrosis

Renal fibrosis is a strong example of the TGF-β–miR-29b–collagen axis [46,66]. In kidney injury models, TGF-β suppresses miR-29b in renal cells, including tubular and mesangial contexts [46,66,92]. This reduction is associated with increased expression of collagen I and collagen IV, two major matrix components involved in renal fibrotic remodelling [10,66]. The renal evidence therefore links the regulatory mechanism described earlier with the collagen targets described in Section 4 [46,66]. miR-29b restoration reduces renal matrix gene expression in experimental systems [46,66,92]. Overexpression of miR-29b represses *COL1A1* and *COL4A1*, including reporter-based support for direct regulation [46,66]. In renal fibrosis models such as unilateral ureteral obstruction and diabetic nephropathy, miR-29b restoration or miR-29-based intervention reduces collagen accumulation and fibrosis-associated outcomes [46,92]. Human chronic kidney disease tissue has also been reported to show reduced miR-29b with increased collagen expression, although this type of patient evidence is mainly correlative [10,66].

The renal evidence is therefore best interpreted as strong mechanistic and preclinical support [46,66,92]. It shows that miR-29b is closely linked to TGF-β-driven matrix regulation in kidney fibrosis [10,46].

However, as with liver fibrosis, translation into human therapy depends on delivery, safety and disease-stage considerations [2,7]. Kidney fibrosis is often chronic and progressive, so any miR-29b-based therapy would need sustained or repeated delivery without impairing normal matrix maintenance [2,10].

### 6.4. Cardiac Fibrosis

Cardiac fibrosis is an important but more complex area of miR-29 biology [27,48,93]. After myocardial injury, miR-29 family expression changes in the heart, and reduced miR-29b has been linked to increased cardiac collagen expression [27,47]. This is consistent with the general model in which miR-29b restrains ECM production [27,29]. Several studies suggest that restoring miR-29b can reduce interstitial collagen deposition and improve aspects of cardiac remodelling in experimental models [27,94].

However, the cardiac literature also includes conflicting evidence [48,49,93]. In pressure overload models of cardiac hypertrophy, partial deletion of both miR-29 gene clusters or pharmacological inhibition of all three miR-29 family members using locked nucleic acid antimiRs protected mice from hypertrophy and fibrosis, improved cardiac function and suppressed pathological gene expression [48]. These findings contrasted with earlier studies where cholesterol-conjugated antagomirs targeting miR-29b increased cardiac collagen expression and perivascular fibrosis [27,48]. Differences in antisense chemistry and targeting of the entire miR-29 family versus individual isoforms may explain these opposing outcomes [48,49,93]. Cardiac remodelling involves multiple cell types, including fibroblasts, cardiomyocytes, endothelial cells and immune cells, and miR-29b may have different effects depending on disease model, timing, target spectrum and cell type [47,48].

Cardiac fibrosis should therefore be presented as context-dependent [48,49,93]. The evidence supports a role for miR-29b in regulating cardiac ECM, but therapeutic manipulation may be more complex than in skin or some lung models [27,48]. Increasing miR-29b could reduce collagen deposition in some settings, but it may also affect protective repair, cardiomyocyte survival or adaptive remodelling depending on timing and dose [48,49,93].

### 6.5. Skin Fibrosis and Systemic Sclerosis

Skin fibrosis provides the strongest clinical proof-of-concept for miR-29 replacement [33,35]. Dermal fibroblasts from systemic sclerosis and fibrotic skin contexts show reduced miR-29 family expression with increased collagen production [33,95]. This pattern is consistent with the wider fibrotic model, but the skin field is more advanced because it includes direct human intervention data [35]. miR-29b mimics reduce collagen expression and fibroplasia in skin fibrosis models [35]. In a human skin wound model, intradermal delivery of a miR-29b mimic reduced collagen gene expression and scar-associated fibroplasia [35]. This is significant because it shows that miR-29 mimic therapy can produce measurable matrix effects in human tissue, not only in cells or animal models [35].

This does not prove that miR-29b therapy will work equally well in all fibrotic diseases [2,7]. Skin is relatively accessible compared with internal organs, and local delivery is more straightforward than delivery to lung, liver, kidney or heart [35,76]. Nevertheless, the skin data provides an important proof-of-concept: restoring miR-29 activity can reduce ECM expression in humans [35]. For this reason, skin fibrosis should be treated as the most clinically advanced evidence base for miR-29b [30,35].

### 6.6. Ocular Fibrosis and Glaucoma-Relevant Tissues

Ocular tissues are an emerging area for miR-29b research [32,65,75,96]. Recent miRNA-mRNA interactome analysis of TGF-β1-treated human trabecular meshwork cells further supports the relevance of miRNA-regulated networks in glaucoma-associated ECM biology [96]. Fibrosis-like ECM remodelling is relevant to several ocular diseases, including glaucoma, neovascular age-related macular degeneration (AMD), proliferative vitreoretinopathy and proliferative diabetic retinopathy [82,97,98,99,100]. In glaucoma, the trabecular meshwork and conventional outflow pathway remain the main focus because ECM accumulation, tissue stiffness and altered cell–matrix signalling can increase resistance to aqueous humour outflow and intra-ocular pressure [101,102,103]. These processes overlap conceptually with the matrix-remodelling mechanisms regulated by miR-29b in other tissues [32,65,75]. Raised intra-ocular pressure is currently the only clinically modifiable risk factor for glaucoma [104].

The rationale for studying miR-29b in ocular fibrosis is therefore strong [32,65,75]. miR-29b targets collagen, matrix maturation and adhesion-related genes that are relevant to trabecular meshwork biology [32,65,75]. If miR-29b is reduced in fibrotic or TGF-β-stimulated trabecular meshwork cells, its restoration could theoretically reduce ECM accumulation and improve matrix balance in the outflow pathway [65,105]. However, compared with lung, skin, liver and kidney fibrosis, direct miR-29b evidence in glaucoma-relevant tissues is less developed [32,105].

Ocular fibrosis should therefore be framed as a biologically plausible but emerging application [32,98]. The target profile of miR-29b supports further investigation, especially in TGF-β-driven trabecular meshwork models and perfused anterior segment systems [65,97,105]. Non-viral nucleic-acid delivery to anterior-eye tissues is supported by work showing topical siRNA delivery to the cornea and anterior eye using hybrid silicon lipid nanoparticles [106]. Future ocular work could also examine posterior segment fibrotic settings where retinal pigment epithelium (RPE) epithelial-to-mesenchymal transition, fibrovascular membrane formation or retinal fibrosis are prominent, including neovascular AMD, proliferative vitreoretinopathy and proliferative diabetic retinopathy [82,99,100]. However, glaucoma-relevant outflow tissues should remain the central ocular focus, and ocular fibrosis should not be presented as having the same level of evidence as pulmonary or skin fibrosis [32,35,76]. Future studies need to establish whether miR-29b is consistently altered in glaucoma-relevant outflow tissues, whether restoring miR-29b reduces pathological matrix deposition, and whether this produces functional effects on outflow resistance or intraocular pressure [65,75,105].

### 6.7. Cross-Organ Synthesis

Across disease models, the evidence supports a consistent but not uniform role for miR-29b in fibrosis [29,30]. The common pattern is reduced miR-29b or miR-29 family expression during fibrotic activation, followed by increased expression of ECM and matrix-associated genes [28,33,66,68].

Restoring miR-29b often reverses part of this programme, reducing collagen expression, fibroblast activation markers and tissue fibrosis in experimental models [31,35,74].

The strongest translational evidence currently comes from skin, where remlarsen/MRG-201 has shown measurable effects on collagen expression and scar-associated fibroplasia in human tissue [35]. Pulmonary fibrosis also has strong preclinical and translational momentum, supported by lung fibroblast data, animal fibrosis models and development of targeted or inhaled mimic approaches [31,76]. Liver and kidney fibrosis provide strong mechanistic and preclinical evidence, especially through hepatic stellate cell activation and TGF-β-driven renal collagen regulation [46,66,68,74]. Cardiac fibrosis remains more context-dependent because both miR-29 restoration and miR-29 inhibition have been reported as beneficial in different models [27,48,94]. Ocular fibrosis is a rational emerging area, but direct disease-specific evidence remains limited [32,65,75,105]. A cross-organ comparison is summarised in Table 4.

## 7. Therapeutic Replacement and Delivery

Therapeutic restoration of miR-29b must overcome the inherent instability of naked RNA, achieve delivery into fibrotic cells, and minimise off-target effects [107,108]. The strategies developed so far blend chemical modifications to increase mimic stability with delivery vehicles that target relevant tissues [107,109].

### 7.1. Mimic Design and Chemical Modification

Synthetic or exogenously delivered miRNA mimics can activate innate immune sensors depending on sequence, chemistry, duplex structure, impurities, dose and delivery vehicle [110,111]. Phosphorothioate linkages increase nuclease resistance, while 2′-O-methyl and 2′-fluoro substitutions can improve stability and reduce toll-like receptor recognition [111,112,113,114]. Locked nucleic acids increase affinity and duplex stability, although the position and number of modified residues must be balanced against altered strand selection and toxicity [112,114]. Therapeutic miRNA mimics are commonly designed as guide/passenger duplexes that favour loading of the intended guide strand into RISC. An agomir is an in vivo-optimised, chemically stabilised miRNA mimic and is often also a duplex; the term should not be used as a synonym for every culture-grade mimic or for a single-stranded construct. MRG-201 used cholesterol conjugation, whereas MRG-229 adds stabilising sugar modifications and a PDGFRβ-binding bicyclic peptide [35,76].

### 7.2. Delivery Platforms, Tissue Targeting and Pharmacokinetics

Even well-designed mimics require carriers to reach target cells [108,109]. Non-viral systems dominate miRNA delivery because they elicit less immune activation than viral vectors [109,115]. Lipid nanoparticles (LNPs) encapsulate the mimic with ionisable cationic lipids and helper lipids such as cholesterol or 1,2-dioleoyl-sn-glycero-3-phosphoethanolamine (DOPE) [116,117]. Polyethylene glycol (PEG) coatings reduce aggregation and prolong circulation [117,118]. Nanostructured lipid carriers, which mix solid and liquid lipids, achieve similar aims and are advancing from preclinical stages [119,120]. Polymeric carriers, including poly(lactic-co-glycolic acid) (PLGA)-based systems and other biodegradable polymeric nanoparticles, protect mimics and permit controlled release [109,121]. Polymersome systems are also relevant to this delivery discussion because amphiphilic polymer vesicles can improve formulation stability, cargo flexibility and delivery of both hydrophilic and hydrophobic therapeutic payloads [122,123]. Other approaches include cell-penetrating peptides, such as the PDGFRβ-binding peptide used in MRG-229, and extracellular vesicles; the latter benefit from low immunogenicity but pose challenges for scalable production [76,124]. The major delivery platforms for miR-29b mimic therapy, including their main advantages and limitations, are summarised in Table 5.

Viral vectors such as adeno-associated virus (AAV), adenovirus and lentivirus, can deliver sustained miRNA expression but carry greater immunogenicity and are more often used for antimiR strategies [108,109].

Because miR-29b suppresses many ECM genes, systemic exposure could impair normal wound healing [1,22]. Targeted delivery therefore seeks to concentrate the mimic in fibrotic tissues [107,108]. Conjugation to receptor-binding peptides is one approach: MRG-229 uses a bicyclic peptide that binds the PDGFRβ, which is abundant on activated fibroblasts [76]. This design allows subcutaneous administration and produces anti-fibrotic effects at doses ten-fold lower than MRG-201 [76]. For liver-targeting, tri-antennary N-acetylgalactosamine (GalNAc) ligands exploit the asialoglycoprotein receptor on hepatocytes; GalNAc-conjugated anti-miR programmes illustrate how this strategy could be adapted for miR-29b [126,127]. Aptamer–miRNA conjugates use RNA sequences that bind cell-surface proteins to direct delivery; aptamer–miR-34c constructs have been explored for lung cancer [128,129]. Local administration can also enhance specificity: intradermal injection of MRG-201 in a human skin model reduced collagen expression and fibroplasia [35], while inhaled or intratracheal routes are under study for pulmonary fibrosis [76,87].

Delivery and pharmacokinetics cannot be separated: particle composition or conjugate chemistry determines serum stability, protein binding, organ distribution, cellular uptake, endosomal escape, intracellular persistence, and clearance [107,108,109]. Local administration to skin, tendon or the eye can reduce systemic exposure, whereas lung, liver, kidney and heart applications require organ- and cell-selective uptake. Demonstrating bulk-organ accumulation is insufficient if the mimic does not reach the activated stromal population.

### 7.3. Comparative and Emerging Delivery Technologies

Each delivery strategy offers trade-offs [107,108]. LNPs have benefited from clinical experience in small interfering RNA (siRNA) therapy but can provoke complement activation and require organ-specific targeting; polymeric carriers allow controlled release but sometimes have lower transfection efficiency [118,121,125]. Polymersome studies further show that polymeric vesicles can support triggered-release monitoring and combination-payload loading, although these remain platform data rather than miR-29b-specific delivery studies [131,132].

Peptide conjugates provide receptor-specific uptake but may trigger immunogenicity; GalNAc conjugates efficiently target hepatocytes but confine applications to the liver; aptamer conjugates offer customisability but face challenges in stability and manufacturing [107,126,128]. Exosomes promise endogenous tropism and low immunogenicity yet remain difficult to scale [108,124]. Engineered exosomes loaded with miR-29b and decorated with targeting ligands may combine endogenous tropism with specificity [124,130]. Clustered regularly interspaced short palindromic repeats (CRISPR)-based systems could up-regulate endogenous miR-29b rather than deliver synthetic mimics, but such approaches remain in their early stages [133,134].

Emerging technologies seek to refine these platforms [108,135]. Stimuli-responsive nanoparticles release cargo in response to pH or redox cues, potentially limiting exposure to non-fibrotic tissues [135,136].

## 8. Clinical Translation

Preclinical activity is not equivalent to clinical efficacy. Human translation requires confirmation of target engagement in the intended cell population, a plausible dose–exposure relationship, durable functional benefit and an acceptable safety margin [107,115,137,138].

### 8.1. Status of miR-29-Family Mimic Programmes

Remlarsen (MRG-201) is a chemically modified double-stranded miR-29b mimic evaluated by local intradermal administration. Human wound studies have shown reduced collagen expression and fibroplasia, establishing target engagement and biological proof-of-concept [35]. A Phase II cutaneous fibrosis study has been completed, but peer-reviewed evidence of durable clinical efficacy has not established remlarsen as an approved therapy. The next-generation peptide-conjugated miR-29 mimic MRG-229 has shown activity in human lung fibroblasts, precision-cut lung slices and bleomycin models, with supportive animal toxicology; published evidence remains preclinical and no human efficacy data are available [76].

TenoMiR (CWT-001) is a proprietary miR-29a mimic (not a miR-29b mimic) delivered locally for lateral epicondylitis [139,140,141]. A first-in-human dose-escalation study reported acceptable tolerability and imaging evidence of tendon remodelling, and a Phase II study has been registered and completed. Peer-reviewed Phase II results are awaited [139,142]. TenoMiR supports the clinical tractability of local miR-29-family replacement, but it must not be presented as miR-29b-specific anti-fibrotic efficacy.

### 8.2. Lessons from Other miRNA Therapeutics

Experiences with other miRNA-directed agents define the translational boundaries for miR-29b [138,143]. Miravirsen, an LNA-modified inhibitor of miR-122, produced prolonged antiviral activity, showing that endogenous miRNA pathways can be modulated clinically [144]. In contrast, the liposomal miR-34a mimic MRX34 was terminated after serious immune-mediated toxicities, illustrating how sequence, dose and carrier can interact [145,146]. Cobomarsen and CDR132L further show that target biology, chemistry, route and indication determine clinical viability [147,148,149,150,151]. These examples support development of miR-29b, while supporting the arguments against class-wide assumptions of efficacy or safety.

## 9. Safety, Off-Target Effects and Translational Considerations

Safety concerns fall into three related categories: on-target effects outside diseased tissue, unintended seed-mediated repression, and formulation- or RNA-sensing toxicity. Because miR-29b normally regulates ECM homeostasis, excessive or systemic activity could weaken normal repair, alter basement membranes or disturb vascular and immune functions [22,83,152,153]. Tissue targeting reduces but does not eliminate this risk because receptor expression and biodistribution change with disease stage.

Synthetic duplex RNA can activate toll-like receptors, RIG-I, MDA5 or PKR depending on sequence, terminal chemistry, impurities, duplex structure and dose [110,111,154,155,156]. 2′-O-methyl, 2′-fluoro and selected LNA modifications can reduce innate recognition, but changes that improve stability may also prolong unintended activity [111,112,114]. Cationic or ionisable lipid systems can contribute complement activation, hepatic exposure or inflammatory responses, whereas peptide and viral systems introduce distinct immunogenicity and reversibility concerns [108,125,157].

A translational safety package should therefore include dose-ranging and recovery periods; tissue and cell-level biodistribution; cytokine, complement, liver, kidney and haematological monitoring; transcriptome-wide off-target assessment; histological evaluation of normal repair; and chronic repeat-dose studies. Duration of RISC loading and reversibility are especially important because fibrosis often requires long-term treatment. Local or activated-fibroblast-enriched delivery are preferable when they provide adequate exposure, but each route requires disease-specific pharmacokinetic and pharmacodynamic validation.

## 10. Controversies and Knowledge Gaps

Despite strong preclinical evidence that restoring miR-29b suppresses fibrosis, the literature supports a cautious, context-specific interpretation rather than a single universal model [29,30,76]. The most obvious example is cardiac remodelling, where miR-29 restoration and miR-29 inhibition have each been associated with benefits in different experimental contexts [27,48,49,93]. This variability likely reflects differences in disease model, timing, oligonucleotide chemistry, isoform specificity and target cell population rather than a simple contradiction in the anti-fibrotic concept [48,49].

These observations highlight the need to define where, when and how miR-29b should be modulated, especially in tissues where matrix repair and pathological fibrosis overlap [27,48,93].

Family-member specificity remains unresolved in much of the literature. miR-29a, miR-29b and miR-29c share the same seed but differ in abundance, stability and subcellular localisation [47,55,64]. Results labelled ‘miR-29′ may therefore support a shared target programme without proving miR-29b-specific efficacy. Conversely, manipulating all family members can produce phenotypes that differ from selective restoration of miR-29b-3p.

Species and tissue differences also complicate the translation of miR-29 research [47,54]. miR-29 sequences are highly conserved across humans, mice and rats, but their regulation varies between organs and developmental stages [30,47]. For instance, the cardiac studies cited above reported age- and stress-dependent fluctuations in miR-29 expression and differences in expression between cardiomyocytes and fibroblasts [27,48]. These observations caution against assuming that mouse data will always predict human outcomes and underscore the need for studies in diverse cell types, including hepatic stellate cells, renal fibroblasts, epithelial cells and smooth-muscle-like cells, and across sexes and ages [54,158,159].

The true spectrum of direct miR-29b targets is also uncertain [22,23,61]. Expression changes after mimic treatment combine direct RISC-mediated repression with secondary effects on cell state and signalling. Standard reporter assays may be performed in heterologous cells, while CLIP approaches can miss interactions because of analytical thresholds or low transcript abundance [160,161]. Directness, disease cell relevance and phenotypic necessity therefore remain separate questions.

Whether miR-29b levels can serve as biomarkers for disease severity or therapeutic response remains unresolved [76,162]. Circulating miR-29 levels are reduced in several fibrotic diseases and, in idiopathic pulmonary fibrosis, lower miR-29 levels have been reported to correlate with increased mortality in patient cohorts [76,85]. However, such associations are correlative and may not hold across populations; further work is needed to define reference ranges, control for confounding variables and determine whether miR-29 changes precede disease progression [107,162]. Individual variability in miR-29 expression, driven by genetics, environmental exposures and co-morbidities, also suggests that patient stratification may be necessary when designing miR-29-based therapies [107,115].

Finally, established scars may respond differently to early active fibrosis. A dose that suppresses pathological matrix deposition during active disease may impair necessary repair after acute injury. Biomarkers that identify active miR-29-responsive disease rather than accumulated inert scar tissue alone have not yet been validated.

## 11. Future Directions

Future work should prioritise causal target mapping in the cells that actually receive therapy. Argonaute CLIP, microCLIP, agoTRIBE and orthogonal reporter/rescue experiments can distinguish direct binding from secondary regulation [160,161,163,164]. Single-cell and spatial profiling should then determine which stromal, epithelial, endothelial and immune populations express miR-29b, its targets, and delivery receptors at different disease stages.

Human organoids, precision-cut tissue slices, perfused organs and ex vivo disease models provide bridges between monoculture and animal studies. For ocular applications, perfused anterior segments could test whether miR-29b changes ECM abundance, tissue stiffness, outflow facility and pressure-related phenotypes rather than molecular markers alone. Comparable functional endpoints are required in lung, liver, kidney, heart and skin.

Therapeutic development should compare local, organ-targeted and activated-fibroblast-targeted constructs using matched pharmacokinetic and pharmacodynamic endpoints. Adaptive dosing may be needed to restore miR-29b towards a physiological range rather than maximise expression. Predictive biomarkers could combine tissue or circulating miR-29, ECM-turnover products, imaging, and cell-state signatures.

Combination strategies with approved anti-fibrotic agents, immunomodulators or therapies that reduce mechanical stress are rational because they address distinct components of the feed-forward loop [115,143]. However, combinations require formal interaction studies and long-term safety assessment. Standardised reporting of family member, strand, chemistry, dose, delivery efficiency, biological replicate number and target-validation level would substantially improve comparability across studies.

## 12. Conclusions

Fibrosis represents the persistence of a normally adaptive repair programme. When inflammation and stromal activation fail to resolve, ECM deposition, collagen maturation, crosslinking and tissue stiffness reinforce one another. miRNAs regulate this transition at the network level, and the miR-29 family occupies a central anti-fibrotic position.

miR-29b represses validated structural and matrix maturation targets, while also altering fibrogenic cell state. HSP47 and LOX illustrate direct control of collagen folding and crosslinking; ADAMTS2 illustrates why a regulated transcript should not be called a direct target without binding-site evidence. Epithelial and endothelial findings broaden the cellular framework but remain more context-specific than the fibroblast evidence.

Skin provides human target-engagement proof-of-concept, lung has advanced preclinical evidence, and liver and kidney provide strong mechanistic support. Cardiac effects remain context dependent, and ocular fibrosis is an emerging application requiring functional validation. Translation will depend on selective delivery, physiological dose restoration, rigorous target assignment and demonstration that chronic treatment reduces pathological scarring without compromising normal tissue repair.

## Figures and Tables

**Table 1:** Comparative framework for physiological wound healing and pathological fibrosis. The categories describe prevailing features; individual tissues may show intermediate or mixed states. Abbreviation: ECM—extracellular matrix.

Process	Physiological Repair	Pathological Fibrosis	Regulatory Consequence
Inflammation	Transient and self-limiting	Persistent or recurrent	Continued cytokine and growth-factor input
Fibroblast activation	Temporary and spatially restricted	Sustained, expanded and mechanically reinforced	Persistent myofibroblast phenotype
ECM production	Provisional matrix matched to repair needs	Production exceeds degradation	Progressive matrix accumulation
Myofibroblast fate	Apoptosis, senescence or deactivation after closure	Survival and continued contractility	Ongoing matrix secretion and contraction
Matrix remodelling	Reorganisation restores architecture and function	Crosslinked, stiff and disorganised scar	Feed-forward mechanotransduction
miRNA state	Expression trends back towards homeostasis	Sustained pro-fibrotic/anti-fibrotic imbalance	Network-level persistence of fibrogenic gene expression

**Table 2:** Functional classification of fibrosis-associated miRNAs. Representative examples are included to position the miR-29 family within the wider regulatory network; classification remains cell-, tissue- and stage-dependent.

Functional Class	Principal Effect	Representative miRNAs	Illustrative Processes and Evidence
Pro-fibrotic fibro-miRs	Promote or stabilise fibrosis	miR-21; miR-199a-5p	Amplification of TGF-β activity, fibroblast activation and repression of anti-fibrotic targets [43,45]
Anti-fibrotic miRNAs	Restrict fibrogenic programmes	miR-29 family; miR-200 family	Direct ECM repression and limitation of TGF-β-dependent EMT or matrix production [27,28,33,44,46]
Context-dependent regulators	Effect varies with compartment, timing or target spectrum	miR-29 family in cardiac remodelling; other tissue-enriched miRNAs	Opposing outcomes can arise after family-wide versus isoform-specific manipulation or in different target cells [47,48,49]

**Table 3:** Selected miR-29b and miR-29-family targets organised by functional category, biological role, family-member specificity and evidence strength. ‘Direct canonical’ requires functional target-site evidence; ‘experimentally supported’ indicates regulation after miRNA manipulation without complete site validation; ‘reported association’ denotes weaker or family-level evidence.

Target	Functional Category	Fibrosis-Relevant Role	miR-29 Scope	Direct 3′UTR Evidence	Model/Validation	Evidence Label
*COL1A1*	ECM structure	Major fibrillar scar collagen	miR-29b	Reporter/target-site evidence reported	Human hepatic stellate cells; mRNA/protein repression	Direct canonical [77]
*COL4A1*	ECM structure	Basement-membrane remodelling	miR-29 family/miR-29b relevant	Reporter evidence	Renal and hepatic contexts	Direct, model-dependent [66,69]
HSP47/*SERPINH1*	Collagen folding	ER chaperone for triple-helix folding and secretion	miR-29b	Full-length 3′UTR reporter; site deletion	LX-2 hepatic stellate cells; mRNA/protein	Direct canonical [37]
LOX	Matrix crosslinking	Covalent collagen/elastin crosslink formation	miR-29b	Full-length 3′UTR reporter; site deletion	LX-2 cells; collagen ultrastructure	Direct canonical [37]
LOXL2	Matrix crosslinking	Collagen crosslinking and tissue stiffness	Mainly family/miR-29a evidence	Reporter evidence in non-fibrotic target cells	Renal/lung cancer-associated models	Direct at family level; extrapolate cautiously [79,80]
ADAMTS2	Procollagen processing	N-propeptide removal before fibril assembly	miR-29 family	No definitive validation in fibrosis	Pulmonary fibroblast transcript profiling	Reported association [28]
*FN1*	Matrix assembly	Fibronectin scaffold and pro-fibrotic signalling	miR-29b	Not fully established	Hepatic stellate-cell mimic studies	Experimentally supported [78]
*ITGB1*; *DDR2*; PDGFRβ	Adhesion/activation	Matrix sensing, migration, proliferation and survival	miR-29b/family	Variable or incomplete	Hepatic stellate-cell expression studies	Supported/indirect components [78,81]
*ELN*; *SPARC*	Elastic/matricellular ECM	Matrix architecture and cell-matrix interaction	Mostly family-level	Variable	Multiple stromal and ocular models	Supported; context-dependent [27,28,65]
*AKT2*	Epithelial plasticity	TGF-β1-associated EMT signalling	miR-29b	Wild-type/mutant reporter support	Retinal pigment epithelial cells	Direct, epithelial context [82]
*VEGFA*; *PDGFB*	Endothelial signalling	Proliferation, angiogenesis and paracrine signalling	miR-29b-3p	Wild-type/mutant reporter support	Retinal microvascular endothelial cells	Direct, non-fibrosis-specific context [83]

**Table 4:** Organ-specific evidence for miR-29b or miR-29-family dysregulation and replacement. The table distinguishes cell type, experimental level and miR-29b specificity so that family-wide observations are not presented as isoform-specific proof.

Organ/System	Cell Type/Model	Observed Change and Scope	Principal Targets/Phenotypes	Intervention	Outcome	Translational Interpretation
Lung	Human lung fibroblasts; IPF tissue; bleomycin and silica models	miR-29 family and miR-29b reduced	Collagens, α-SMA, ECM gene programme	Mimic, agomir or peptide-targeted mimic	Reduced collagen and histological fibrosis	Strong preclinical; no established clinical efficacy [28,31,76,84,87,88]
Liver	Human/rodent hepatic stellate cells; CCl4 and bile-duct ligation	miR-29b/family reduced during activation	*COL1A1*/2, *FN1*, HSP47, LOX, PI3K/AKT	Overexpression or vector delivery	Reduced activation and matrix; apoptosis in selected models	Strong mechanistic and preclinical [37,68,74,77,78]
Kidney	Tubular and mesangial cells; UUO; diabetic nephropathy	TGF-β/Smad3 suppresses miR-29	Collagen I/IV and fibrotic markers	miR-29b restoration	Reduced matrix accumulation	Strong mechanistic/preclinical; human data mainly correlative [46,66,92]
Heart	Myocardial infarction and pressure-overload models	Cell-, stage- and family-dependent	Collagens, TGF-β/Smad and Wnt-associated programmes	Restoration or family-wide inhibition	Opposing outcomes across models	Context-dependent; requires cell-specific validation [27,48,49,93,94]
Skin	Systemic-sclerosis fibroblasts; human incisional wound model	miR-29 reduced in fibrotic fibroblasts	Collagen and fibroplasia	Local remlarsen/MRG-201	Reduced ECM expression and fibroplasia	Strongest human proof-of-concept, not broad clinical efficacy [33,35]
Eye	Trabecular meshwork; RPE and retinal endothelial models	TM dysregulation plausible; direct disease evidence limited	ECM; *AKT2*; *VEGFA*/*PDGFB*	Mainly in vitro or proposed local replacement	ECM/EMT/angiogenic effects; outflow benefit untested	Emerging; functional perfusion and IOP studies needed [65,75,82,83,105]

**Table 5:** Delivery strategies relevant to miR-29b replacement. Advantages and limitations refer to platform-level evidence unless miR-29b-specific data are stated.

Strategy	Main Features	Advantages	Limitations
Lipid nanoparticles	Ionisable lipids with helper lipids and PEG coatings [116,117]	Efficient encapsulation and endosomal escape; clinical platform experience	Complement or innate immune activation; organ targeting required [118,125]
Nanostructured lipid carriers	Solid/liquid lipid mixtures [119,120]	Potential high loading and formulation flexibility	Predominantly preclinical; stability and reproducibility
Polymeric nanoparticles/polymersomes	Biodegradable matrices or amphiphilic vesicles [109,121,122,123]	Controlled release and cargo flexibility	Variable transfection and manufacturing complexity
Peptide conjugates	Receptor-binding moiety attached to mimic; BiPPB in MRG-229 [76]	Cell-enriched uptake and improved potency	Receptor heterogeneity and possible immunogenicity
GalNAc conjugates	Tri-antennary ligands for hepatocyte ASGPR [126,127]	Highly efficient liver delivery	Targets hepatocytes rather than all liver fibrogenic cells
Aptamer conjugates	Cell-surface binding RNA ligands [128,129]	Customisable targeting	Stability, synthesis and scale-up
Extracellular vesicles	Natural or engineered vesicles [124,130]	Potential endogenous tropism and low immunogenicity	Loading consistency, potency and scalable manufacture
Viral vectors	AAV, adenovirus or lentivirus [108,109]	Durable expression and high transduction	Immunogenicity, reversibility and insertional risk vary by platform

## Data Availability

No new data were created or analyzed in this study. Data sharing is not applicable to this article.
